# Ultrasound-Guided Regional Anesthesia–Current Strategies for Enhanced Recovery after Cardiac Surgery

**DOI:** 10.3390/medicina57040312

**Published:** 2021-03-25

**Authors:** Cosmin Balan, Serban-Ion Bubenek-Turconi, Dana Rodica Tomescu, Liana Valeanu

**Affiliations:** 1 1st Department of Cardiovascular Anesthesiology and Intensive Care, “Prof. C. C. Iliescu” Emergency Institute for Cardiovascular Diseases, 258 Fundeni Road, 022328 Bucharest, Romania; bubenek@alsys.ro (S.-I.B.-T.); liana.valeanu@yahoo.com (L.V.); 2 Department of Anesthesiology and Intensive Care, University of Medicine and Pharmacy “Carol Davila”, 8 Eroii Sanitari Blvd, 050474 Bucharest, Romania; 3 3rd Department of Anesthesiology and Intensive Care, Fundeni Clinical Institute, 258 Fundeni Road, 022328 Bucharest, Romania; danatomescu@gmail.com

**Keywords:** cardiac surgery, enhanced recovery, regional anesthesia, ultrasound, paravertebral blocks, fascial plane blocks, nociception level index

## Abstract

With the advent of fast-track pathways after cardiac surgery, there has been a renewed interest in regional anesthesia due to its opioid-sparing effect. This paradigm shift, looking to improve resource allocation efficiency and hasten postoperative extubation and mobilization, has been pursued by nearly every specialty area in surgery. Safety concerns regarding the use of classical neuraxial techniques in anticoagulated patients have tempered the application of regional anesthesia in cardiac surgery. Recently described ultrasound-guided thoracic wall blocks have emerged as valuable alternatives to epidurals and landmark-driven paravertebral and intercostal blocks. These novel procedures enable safe, effective, opioid-free pain control. Although experience within this field is still at an early stage, available evidence indicates that their use is poised to grow and may become integral to enhanced recovery pathways for cardiac surgery patients.

## 1. Introduction

Cardiac surgery (CS) generates a unique set of challenges compared to non-cardiac surgery. Postoperative outcomes and quality of life result from several factors, including demographic characteristics, comorbidities, type and quality of surgical intervention, the extent of the systemic inflammatory response, range of organ dysfunction and pain [1,2,3,4]. Conveniently, many of these factors are amenable to optimization. To this end, enhanced recovery after surgery (ERAS) programs have evolved and are now commanded by a multidisciplinary consensus in CS [5].

Pain management is a crucial element of cardiac ERAS. Adequate analgesia is a prerequisite to ensure patient comfort, low morbidity, early mobilization, and cost effectiveness. Postoperative pain is multifaceted and may result from various interventions, including sternotomy, thoracotomy, chest drains and leg vein harvesting. One study found that maximal pain intensity in CS was usually moderate [6], but severe acute postoperative pain was also reported elsewhere and more frequently associated with chronic post-sternotomy pain [7].

Traditionally, opioids were considered the mainstay for pain management after CS based on a predictable hemodynamic profile. Acknowledged risks associated with their use (e.g., hyperalgesia, opioid dependence, respiratory depression, nausea and vomiting, immunosuppression, ileus, delirium, prolonged postoperative recovery) fueled that which now represents a central tenet in the ERAS paradigm–multimodal analgesia (MA) [8]. MA built on drug combinations is not faultless [9]; N-methyl-D-aspartate (NMDA) antagonists may bring about sympathetic hyperactivity, central alpha-2 agonists can cause bradycardia and hypotension, and nonsteroidal anti-inflammatory agents are associated with renal dysfunction and abnormal clotting.

Regional anesthesia/analgesia (RA) represents a valid alternative for the MA repertoire. It obviates many of the drawbacks of drug-based MA strategies, albeit with its particular challenges [10]. Classical neuraxial techniques such as thoracic epidural anesthesia (TEA) and landmark-based paravertebral blocks (PVB_LM_) constituted the standard regional approach to ensure chest wall pain relief before ultrasound (US) virtually revolutionized RA. Bleeding complications (e.g., spinal epidural hematoma (SEH)) were the primary concern regarding the use of TEA and PVB_LM_ in CS [11]. This may explain to some extent why CS fell behind other surgical specialties regarding the large-scale implementation of ERAS programs. Since its inception, US-guided RA (USRA) has helped improve existing techniques (i.e., PVB) and favored the design of new ones. Specifically, real-time US needle-tracking is essential to perform chest wall fascial plane blocks (CWFPB) [12]. Delivery of local anesthetics (LA) between myofascial layers spares the neuraxium and blocks the nerves as they course within that tissue plane. Reasons for the growing popularity of CWFPB include (1) ease of performance; (2) excellent safety profile; (3) good efficacy in various clinical settings. The scope of this review is to address the use of RA in CS, with particular reference to the indications, techniques, and complications of currently available CWFPB (see Table A1, Appendix A).

## 2. Techniques

RA of the chest wall may be performed at various points along an arch coursing anteriorly from the posterior midline. With TEA as gold standard regarding the breadth of somatic and sympathetic blockade, CWFPB exhibit a variable decrement in their effect as they approach the anterior midline. Autonomic effects are retained proportionally to the extent of LA spread into the epidural space, and the area of sensory loss is inversely related to the distance between the injection spot and spine. A considerable inter-individual variation in the extent and intensity of CWFPB exists, and several reasons may represent the root cause of this: (1) existence of differential sensory blockade [13]; (2) reliance on passive LA spread to achieve analgesia; (3) redundant innervation between peripheral nerve territories, including midline overlapping [14,15].

### 2.1. Thoracic Epidural Anaesthesia (TEA)

The role of TEA in cardiac ERAS programs remains an intensely debated topic. TEA produces robust chest wall pain relief, yet it repeatedly failed to improve perioperative morbidity and mortality in CS populations [16]. Potential reasons include the fact that TEA benefits may have a disproportionate impact on CS pain or because TEA side effects and complications may offset its benefits. Notably, pain associated with CS is typically moderate [6], so less intense analgesia (i.e., CWFPB) might suffice. In contrast, adverse events associated with TEA may be clinically relevant (e.g., respiratory depression with epidural opioids and hypotension with epidural LA) and potentially catastrophic (e.g., SEH).

Cardiac sympatholysis was shown to benefit myocardial blood flow [17] but also blunt the heart capacity to cope with hemodynamic challenges, especially within specific subgroups such as those with established pulmonary hypertension [18].

The calculated maximum risks of SEH in CS after TEA were 1:1500 with 95% confidence and 1:1000 with 99% confidence, respectively [19]. In a recent meta-analysis of over 6000 patients, Landoni et al. estimated this risk at 1:3552 (95% CI 1:2552–1:5841) [20]. Placing the epidural one day before surgery could prevent bleeding complications, but such practice patterns would contradict the very essence of ERAS programs.

Overall, minimization of risks outweighs maximization of analgetic potential. Adequate patient selection, risk factors, and anesthesiologist’s expertise must be carefully balanced before pursuing TEA or any other type of neuraxial technique. Until more evidence becomes available, the risk-benefit ratio of neuraxial analgesia remains prohibitive.

### 2.2. Paravertebral Blocks (PVB)

#### 2.2.1. Mechanism and Clinical Applications

**Figure 1:**
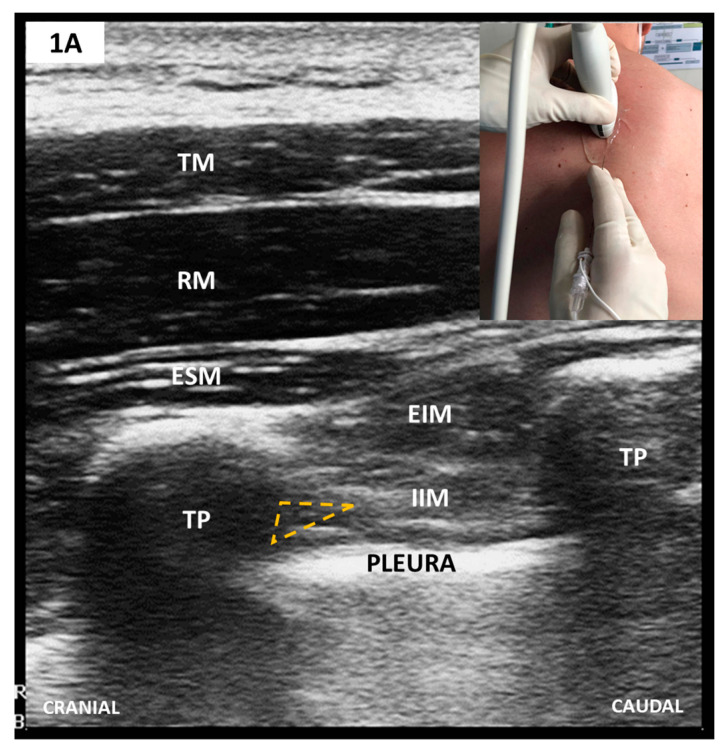

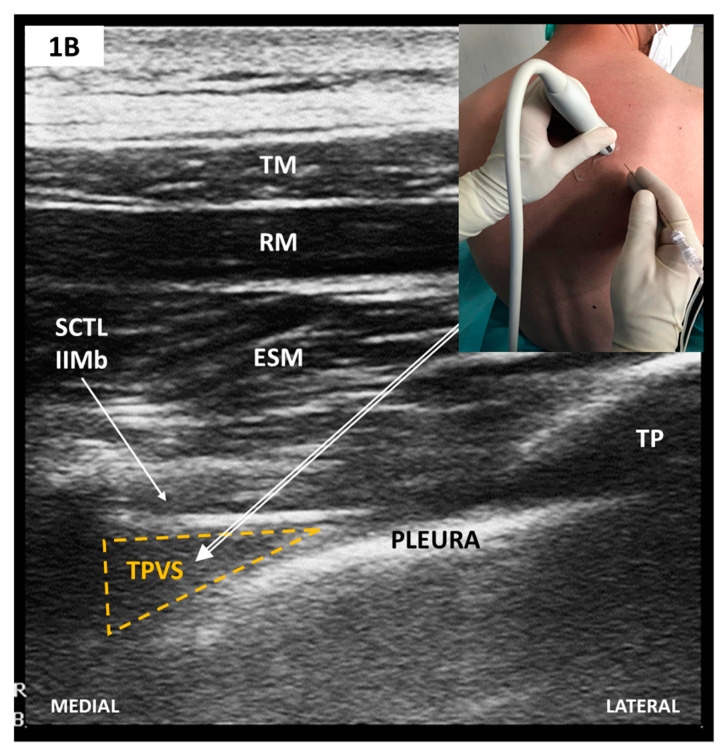
(**A**) Parasagittal scan of thoracic paravertebral space (TPVS); (**B**) Transverse/oblique scan of TPVS after 75-degree anti-clockwise rotation from A. The needle tip’s target is TPVS, which, after probe rotation, appears enlarged and lies anteriorly to superior costotransverse ligament (SCTL)/IIMb (see text).TM, trapezius muscle; RM, rhomboid muscle; ESM, erector spinae muscle; EIM, external intercostal muscle; IIM, internal intercostal muscle; TP, transverse vertebral process; SCTL, superior costotransverse ligament; IIMb, internal intercostal membrane; TPVS, thoracic paravertebral space.

PVB involves LA injection into the thoracic paravertebral space (TPVS) to block the spinal nerve roots as they emerge from the intervertebral foramina. TPVS is a triangular-shaped space on both sides of the vertebrae bounded anterolaterally by the parietal pleura, medially by the posterolateral parts of the vertebral body and posteriorly by the superior costotransverse ligament (SCTL) (see also Figure 1). TPVS communicates laterally with the intercostal space, and medially with the epidural space. TPVS is also contiguous with its contralateral counterpart but to a much lesser extent whereas its cranial extension remains ill-defined. Caudal and rostral segmental spread of the LA drug from the injection site generates multilevel ipsilateral somatic and autonomic blockade, with epidural and intercostal LA dispersions likely contributing substantially to analgesia [21]. The clinical effect of single-level PVB_LM_ is highly variable because the LA spread is unpredictable [22]. Consequently, a multiple-injection technique was commonly considered superior to single-injection patterns [23,24]. This theory was first challenged by Renes et al. [25] and Marhofer et al. [26] who used US-guidance to perform PVB (PVB_US_). Later, Uppal et al. [27] demonstrated that single- and multilevel PVB_US_ are equivalent regarding coverage and pain relief duration. Conveniently, the single-level PVB_US_ are markedly faster and better tolerated by patients, two prerequisites of any ERAS strategy.

Compared to PVB_LM_, PVB_US_ are more reliable and safer [28]. Two assets, equivalent analgesia to TEA but with fewer complications [29,30,31,32,33,34] and unilateral sympathectomy, favored the resurgence of PVB_US_. Still, the latter proves itself ineffectual in CS with sternotomy since this surgery requires bilateral nerve blockade.

As with TEA, hemorrhagic complications represent a crucial factor to consider. In contrast to TEA, risk quantification of SEH after PVB is less evident and intensely debated. The latest American Society of Regional Anesthesia and Pain Medicine (ASRA) Practice Advisory on RA and anticoagulation maintains the same recommendations for PVB as for any other neuraxial block [11]. Equivocally, ASRA does not differentiate between PVB_LM_ and PVB_US_, and between single-shot PVB and PVB with catheters. New data suggests that US guidance during paravertebral blockade could virtually abrogate spinal injury risk even with the large heparin dosing needed in cardiopulmonary bypass (CPB) [35]. El Shora et al. recently compared PVB_US_ with catheter to TEA to manage pain after on-pump CS [36]. Catheters were placed immediately after induction in both study groups, and LA infusion was started only postoperatively. PVB_US_ were non-inferior to TEA regarding pain relief, and bleeding complications were not reported in either group.

Future studies will have to address two aspects to maximize the benefits and minimize the potential risks associated with PVB_US_. The first is concerned with single-shot PVB being safer than PVB with TPVS catheters because catheter misplacement, including epidurally, is still possible even with US [37,38]. The second aspect is concerned with nerve blockade timing, as suggested by Richardson et al. [39]. Compared to PVB_US_ established after surgery, preemptive PVB_US_ may be better tailored to fast-tracking as it would also mitigate the intraoperative opioid consumption.

The best strategy to implement PVB_US_ has yet to be established. Further research is needed before the routine use of paravertebral blockade in CS is either supported or refuted.

##### Sonoanatomy and Block Techniques (Figure 1)

PVB_US_ have superseded PVB_LM_ in every aspect. A comprehensive review described at least nine approaches, all of which share the same three sonoanatomical landmarks circumscribing TPVS—rib, pleura, and transverse process (TP) [40]. At present, formal recommendations on the best way to perform PVB_US_ do not exist. Instead, personal factors relating to skill, experience and perceived safety seem to play a decisive role. An objective comparative evaluation of currently used PVB_US_ techniques is essential to enable an informed PVB-based MA.

TPVS scanning breaks down to 4 elements: (1) plane of US beam orientation (i.e., transversal versus sagittal); (2) needling technique (i.e., out-of-plane versus in-plane); (3) direction of angulation (i.e., lateral versus medial, and caudal versus cranial respectively) and (4) safety limit for needle tip (i.e., anteriorly or posteriorly to SCTL) [40]. Choosing between these elements entails a trade-off between two goals, simplicity and accuracy. The latter is advocated in our institute, so we perform an in-plane, lateral to medial, transversal/oblique approach with a safety limit set anteriorly to SCTL (see Figure 1). Based on currently available evidence, catheters are excluded with CPB heparin dosing.

**Figure 2:**
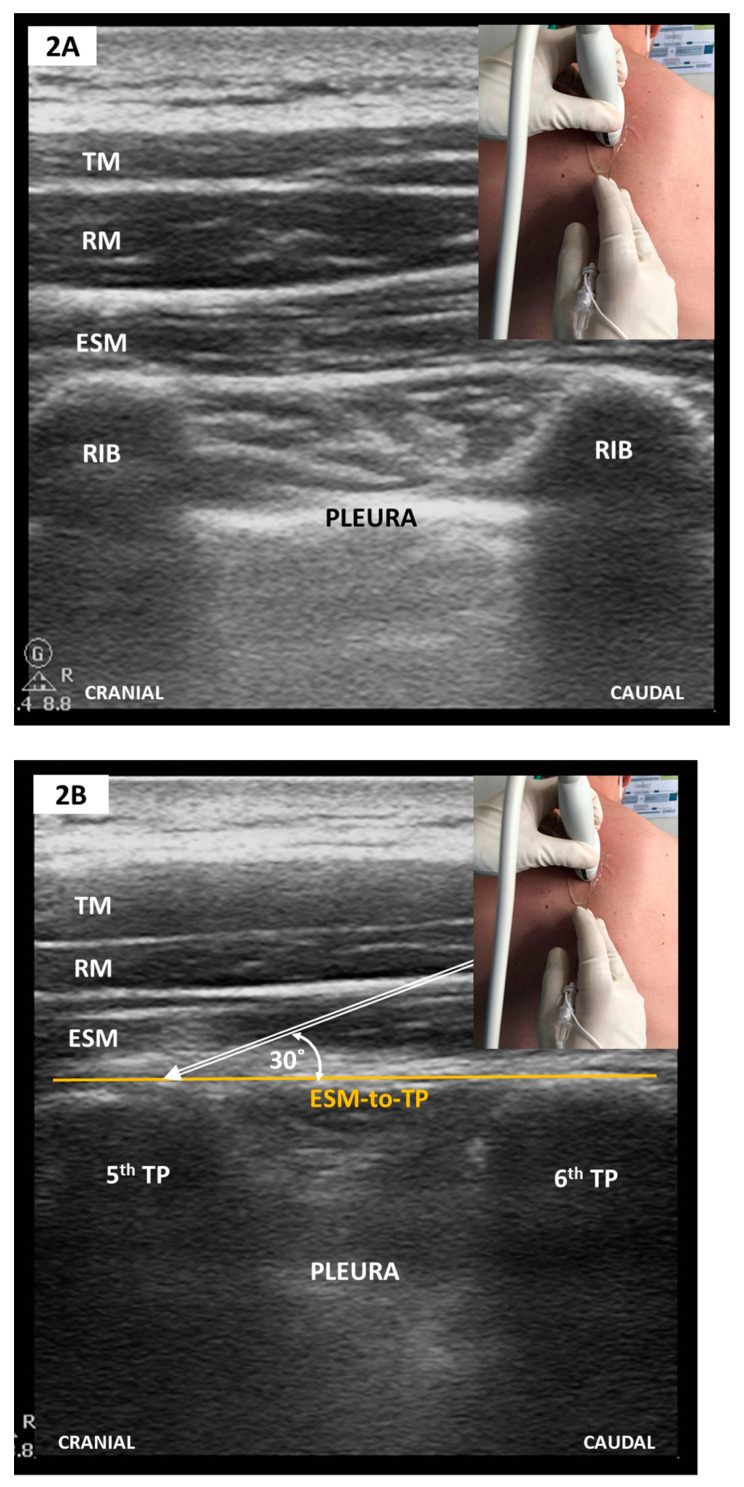
(**A**) Parasagittal scan—rib level; (**B**) Parasagittal scan—TP level (see text). TM, trapezius muscle; RM, rhomboid muscle; ESM, erector spinae muscle; TP, transverse vertebral process; ESM-to-TP, erector spinae muscle -to-transversus process plane.

Scanning starts with the linear-array transducer placed in a parasagittal plane to identify the adjacent TP, recognizable as flat, rectangular hypoechoic structures (see also Figure 2). Anti-clockwise rotation to a transversal/oblique plane displays the TPVS. The needle is inserted in-plane, latero-medially and advanced until it reaches the wedged-shaped TPVS. Adequate LA injection pushes the parietal pleura anteriorly. Preemptive bilateral single-shot blocks are performed at the level of the fourth thoracic vertebrae. This alone may provide intraoperative analgesia long enough to sustain most types of CS.

### 2.3. Chest Wall Fascial Plane Blocks (CWFPB)

#### 2.3.1. Posterior CWFPB-Erector Spinae Plane Block (ESPB) and other PVB Variants

##### Mechanism and Clinical Applications

Post-mortem data challenge the traditional view that TPVS is a discrete anatomical space and suggest that the SCTL is permeable to LA drugs [41]. Hence, paravertebral blockade of nerves could still be elicited by placing the needle tip outside but close enough to the TPVS.

US guidance has facilitated the emergence of several more superficial needle placement techniques, all collectively labelled as “paraspinal blocks” [42] or “PVB by proxy” [43]. These include the retrolaminar block (RLB) [44,45], midpoint transverse process to pleura block (MTPB) [41], intercostal/paraspinal block (ICPB) [46], rhomboid intercostal and sub-serratus block (RISS) [47], and erector spinae plane block (ESPB) [48]. Depending on their underlying pathway of LA spread, these novel blocks produce a variable combination of ipsilateral somatic and autonomic blockades, the extent of which remains open for further research. Amongst them, ESPB is the most well characterized to date.

The ESPB target for LA deposition is the plane between the erector spinae muscle (ESM) and the thoracic TP tip. Correct single-level LA injection should lift the ESM off the TP and allow the ipsilateral craniocaudal volume-dependent [49] LA spread across several contiguous dermatomes (i.e., 3 to 7 intercostal spaces) [50]. As with PVB, transforaminal, intercostal and circumferential epidural diffusions likely contribute to its mechanism of action [50,51].

Krishna et al. compared bilateral single-shot ESPB with control (i.e., general anesthesia alone) in CS with sternotomy and found reduced postoperative pain, time to extubation, time to ambulation, opioid usage and total length of intensive care unit (ICU) stay [52]. Interestingly, rescue analgesia was reported in the intervention group only ten hours after extubation compared to six hours in the control group (*p* = 0.0001). Macaire et al. used a before-and-after design to show that in open CS a preemptive strategy with bilateral ESPB catheters is associated with reduced intra- and postoperative opioid consumption. Consequently, several ERAS endpoints were favorably altered, including postoperative adverse events (hypotension, nausea/vomiting and hyperglycemia) and times to chest tube removal and first mobilization. The authors found no differences in extubation time and pain during the first mobilization. Another RCT showed comparable postoperative pain scores between bilateral continuous ESPB and TEA in 50 patients undergoing open CS [53]. Finally, Bousquet at al. endorse the association of bilateral parasternal block with bilateral ESPB [54] given that ESPB alone may sometimes fail to provide adequate parasternal analgesia [55]. This dual blockade significantly reduced the intraoperative sufentanil and postoperative morphine usage in a 20-patient cohort [54]. These four studies did not report any RA related adverse effects, but then again, neither was appropriately powered to detect them.

Although promising, results from these clinical studies are not generalizable. There is a potential bias concerning the small patient populations, blinding and randomization. Further studies are mandated to fully understand the benefits and extent of incorporating ESPB into routine clinical practice.

##### Sonoanatomy and Block Tachnique (Figure 2)

Scanning starts with the linear-array transducer set 5–6 cm away from the dorsal midline in a parasagittal orientation. The ribs are then displayed as rounded acoustic shadows with an interceding hyperechoic pleural line (see Figure 2A). Sliding the transducer medially along the short axis allows visualization of the TP as flat, squared-off acoustic shadows (see Figure 2B). Additionally, the pleural line is more in-depth and ill-defined. A too medial position identifies the thoracic laminae as a continuous flat hyperechoic line with regularly interspersed notches representing the facet joint interfaces. Needle insertion follows an in-plane approach, either craniocaudal or vice versa, to contact the ESM-to-TP plane. Real-time imaging guarantees correct LA hydro-dissection beneath the ESM and catheter placement whenever continuous pain relief is warranted. Single-level injection ESPBs (i.e., at the 5th thoracic vertebrae), as initially described by Forero et al. [48], continue to be the norm but this view has recently been challenged by Tulgar et al. who propose a bilevel approach to ensure a more homogeneous LA spread [56].

#### 2.3.2. Anterolateral CWFPB—Pectoral Blocks and Serratus Plane Block

##### Mechanism and Clinical Application

**Figure 3:**
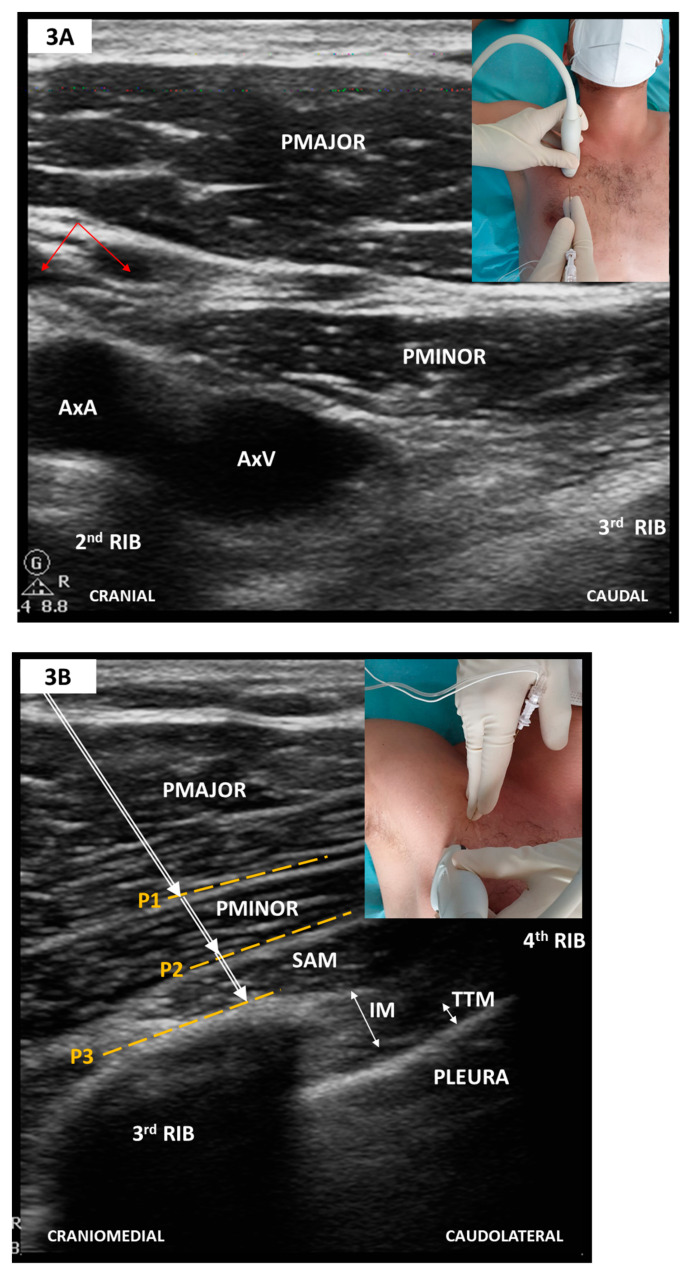
(**A**) Parasagittal scan along the medioclavicular line-2nd rib level; (**B**) Oblique scan after a slight medial tilt with inferolateral sliding towards the midaxillary line-4th rib level (see text). PMAJOR, pectoralis major muscle; PMINOR, pectoralis minor muscle; AxA, axillary artery; AxV, axillary vein; red arrows, thoracoacromial artery and vein; SAM, serratus anterior muscle; IM, intercostal muscle; TTM, transversus thoracic muscle; P1, PECS I plane; P2, superficial plane for SAPB/PECS II; P3, deep plane for SAPB/PECS II. To elicit an adequate SAPB coverage, P2 or P3 need to be targeted at the 4th or 5th rib level.

Anterolateral CWFPB provide ipsilateral somatic anesthesia of the upper anterolateral hemithorax but may spare the anterior branches of the intercostal nerves and hence do not consistently provide anesthetic coverage to the ipsilateral parasternal region [12]. This theoretically hinders their use in CS with sternotomy. Established techniques include the serratus anterior plane block (SAPB) [57] and the pectoralis block type I (PECS I) [58] and II (PECS II) [59]. Whilst PECS I and SAPB are distinct blocks, targeting two separate musculofascial planes, PECS II merely represents an attempt to achieve both PECS I and SAPB during a two-staged single needle pass (see also Figure 3).

A 40-patient RCT compared PECS II with no block as part of a postoperative MA strategy in patients undergoing CS with sternotomy. PECS group patients were extubated earlier, had lower pain scores and fewer episodes of rescue analgesia [60].

SAPB was studied in minimally invasive heart valve surgery (MIHVS) with right thoracotomy and minimally invasive direct coronary artery bypass (MIDCAB) with left thoracotomy. Berthoud et al. compared postoperative single-shot deep SAPB to continuous wound infiltration (CWI) and reported significantly lower morphine consumption, reduced length of ICU stay and improved pain control during the first 48 h following MIHVS [61]. Another group of authors compared pre-incisional single-shot and postoperative catheter-based deep SABP against parenteral morphine [62]. The intraoperative opioid usage remained unaffected, but the combined regional nerve blockade significantly spared the postoperative morphine consumption. Nevertheless, this did not change the postoperative course, that is, ICU and hospital lengths of stay and ventilator-free days. According to one study, SAPB appears well suited for MIDCAB thoracotomies [63] but remains inferior to PVB in terms of analgesic coverage and intensity [64]. Lastly, SAPB and PECS II showed an equivalent analgesic effect in an RCT conducted on pediatric patients undergoing CS with thoracotomy without CPB [65].

Anterolateral CWFPB have an excellent safety profile that will allow their ongoing integration in cardiac ERAS pathways. Their impact relies markedly on adequate timing (i.e., pre- versus postoperative blockade) and indication.

##### Sonoanatomy and Block Technique (Figure 3)

PECS I targets the lateral (C5–C7) and medial (C8–T1) pectoral nerves travelling within the fascial plane between the pectoralis minor and major muscles. SAPB targets the plane either above or below the serratus anterior muscle (SAM). Although some authors favor the latter [66], the differences between these two juxtaposed fascial planes have not yet been elucidated. SAPB blocks the lateral cutaneous branches of the intercostal branches and, when superficially performed, the long thoracic (C5–C7) and thoracodorsal nerves (C6–C8). A single needle pass may secure both blocks (i.e., PECS II) and achieve ipsilateral anesthesia of the anterolateral hemithorax and axilla. Scanning is carried out craniocaudally along the midclavicular line, sliding laterally to intersect the midaxillary line at the fourth and fifth ribs level. Needle insertion follows an in-plane, mediolateral approach (see Figure 3).

#### 2.3.3. Anteromedial CWFPB—Parasternal Block Variants

##### Mechanism and Clinical Applications

These blocks complement the anterolateral CWFPB by providing anesthesia confined to the parasternal region [67]. Depending on where the anterior branches of the intercostal nerves are blocked, anteromedial CWFPB consist of two interrelated approaches: the pecto-intercostal fascial plane block (PIFB) [68] and transverse thoracic muscle plane block (TTMPB) [69]. The former is the injection of LA between the external intercostal and pectoralis major muscles. The latter targets a deeper fascial layer between the inner intercostal and transverse thoracic muscles. Some authors promote PIFB because of a potentially superior safety profile [70,71] and others inform that the transverse thoracic muscles may be too thin to identify with US [72].

Both parasternal variants have been evaluated in CS with sternotomy. Two small RCTs looked at bilateral single-shot PIFB as part of a postoperative MA regimen. Adverse effects were not recorded, and pain scores were significantly reduced in both trials [73,74]. There was a trend towards reduced cumulative opioid consumption, but this reached statistical significance in only one trial [73]. Anecdotal evidence supports the combination of PIFB with other fascial plane blocks as clinically required [75]. Furthermore, such an approach may be readily generalizable to all CWFPB and lend itself to an individualized USRA.

Preemptive single-shot bilateral TTMPB was compared with placebo in an RCT of 48 adult patients undergoing CS with median sternotomy. Several ERAS-specific outcomes were significantly improved, including first 24 h opioid requirement, rescue analgesia, pain scores, and ICU discharge time [76]. Similar findings have been reported by several pediatric RCTs in CS via midline sternotomy [77,78], with one trial using a combination of TTMPB with rectus sheath block [79].

##### Sonoanatomy and Block Technique (Figure 4)

**Figure 4:**
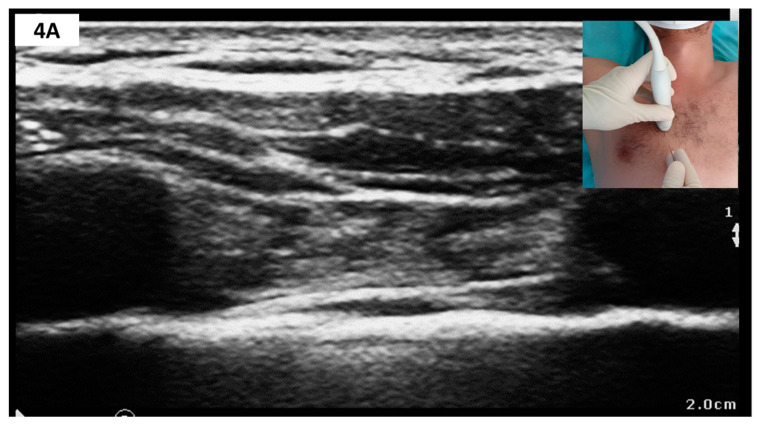

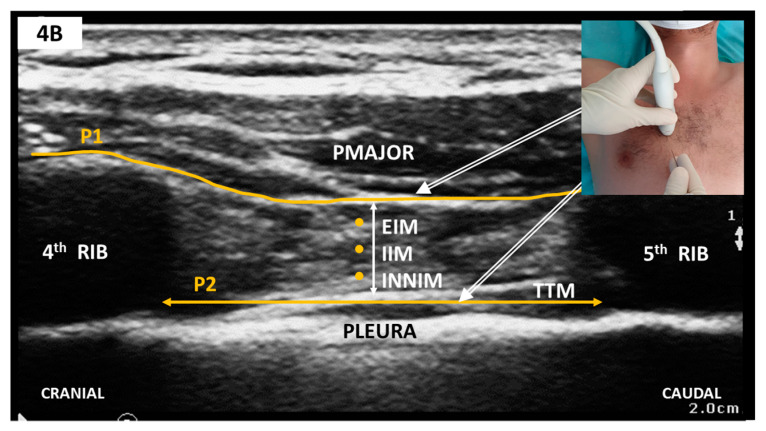
(**A**) Sagittal parasternal scan; (**B**) Sagittal parasternal scan with markings. Note that TTM appears as a hypoechoic band folding over the hyperechoic pleura. PMAJOR, pectoralis major muscle; EIM, external intercostal muscle; IIM, internal intercostal muscle; INNIM, innermost intercostal muscle; TTM, thoracic transversus muscle; P1, target plane for PIFB; P2, target plane for TTMPB.

The linear-array probe is placed in the parasagittal plane, 1 cm lateral from sternum’s edge in the fourth or fifth intercostal space (see Figure 4). Structures to be identified include the pectoralis major muscle, intercostal muscle, thoracic transversus muscles and rib shadows with the intervening pleural line. The internal thoracic artery and vein run longitudinally and share the same plane with TTMPB (i.e., superficial to the thoracic transversus muscle). Perforating branches may cross the intercostal muscles to reach the sternum. Careful scanning in two orthogonal planes is thus mandated before needle insertion to avoid inadvertent vascular puncture. To this end, some authors recommend a transversal approach with lateral to medial needle advancement [72]. Regardless of probe orientation, one or both target planes can then be selected to deposit LA using an in-plane approach.

## 3. Complications

US-assistance has dramatically increased the safety and efficiency of RA techniques resulting in improved outcomes. Reports of complications are scarce and unsystematic. Although local anesthetic systemic toxicity (LAST) is virtually a shared complication of all blocks, it may be more often reported with blocks performed in highly vascular compartments. That was the case with PVB in a case series of eight patients undergoing coronary artery bypass grafting (CABG), where potentially toxic ropivacaine concentrations were reportedly common [80]. Of note, PVB were performed using a landmark technique, and catheters were placed in all patients. Similarly, Lockwood et al. cautioned that systemic absorption after PVB_LM_ is highly probable, especially with continuous catheter infusions [81]. Such findings are compelling enough to consider, regardless of block location and technique, the following precautions: (1) do not exceed the maximum recommended LA dose (see also Table A1); (2) addition of epinephrine to delay systemic absorption; (3) be ready to monitor, recognize and treat LAST; and (4) consider US to enable precise needle advancement [82].

Sympathectomy varies in extent and intensity and is common with posterior nerve blocks, mostly bilateral PVB. Compared to PVB, posterior CWFPB seem less associated with hypotension and bradycardia [83], probably because the epidural spread is lower than initially thought [84].

Performance of PVB and CWFPB can, in theory, result in iatrogenic pneumothorax. Nevertheless, the incidence of this will remain undefined given that chest tubes are invariably present in CS with median sternotomy.

Although PVB are formally contraindicated with CPB anticoagulation regimens, the same recommendations may not apply to the more superficial CWFPB. To date, there are no reported hemorrhagic complications after any of the CWFPB, with anecdotal evidence supporting their use in contexts otherwise prohibitive for classical neuraxial techniques [85].

## 4. Perspective

The best way to provide RA as part of cardiac ERAS strategies has become a topic of considerable interest. Future trials are needed to compare currently available USRA techniques (e.g., PVB versus posterior and anterior CWFPB), establish the optimum time to start the nervous blockade (i.e., pre- versus postoperatively) and understand the role of various perineural adjuvants. This last issue could have momentous consequences as it may enable prolonged duration of single-injection nerve blocks and circumvent the use of catheters [86]. Catheter-free RA is faster to implement, more tolerable and perceivably safer. Furthermore, a simplified technique without additional catheter attempts may promote adherence and widespread use amongst anesthesiologists.

Monitoring regional blockade can be difficult under general anesthesia. With conscious, awake patients, preemptive blockades could be assessed by sensory testing (i.e., pinprick or cold stimulus), but this would delay the operation by at least twenty minutes. Intraoperative nociception monitors could help run an individualized and precise opioid-sparing strategy starting with induction. One trial is currently underway to evaluate the efficacy of ESPB on perioperative opioid consumption in CS with sternotomy during goal-directed anti-nociception using the Nociception Level (NOL) index (NCT04338984).

## 5. Conclusions

USRA favors improved outcomes coupled with an excellent safety profile and has gained considerable momentum in fast-track cardiac surgery over the last decade. Young adults (i.e., mean age 50 years) undergoing elective cardiac surgeries with relatively short aortic cross clamp times seem to derive the greatest benefits, including opioid sparing, reduced time to extubation, earlier mobilization and improved perioperative pain control. Upcoming trials are expected to provide the missing links needed to standardize the integration of RA in cardiac ERAS pathways. Until such time, USRA remains a valuable adjunct in cardiac perioperative care that calls for a personalized application encompassing both anesthesiologist’s expertise and patient’s characteristics.

## Data Availability

Not applicable.

## Appendix A

**Table A1:** Ultrasound-guided regional nerve blocks in cardiac surgery—technical considerations.

Block	Target Plane or Space	Target Nerve	Autonomic Blockade	Maximum Area of Sensory Loss	Surgical Approach—Best Fit	LA Volume for Single-Shot Block/Side #	Practice Patterns
**PVB**	TPVS	Dorsal and ventral rami of spinal nerve roots	Yes	Ipsilateral hemithorax	Sternotomy (BLB)	20–25 mL if single-level (4th TP) or 4–5 mL with multilevel strategy	• Formal contraindication with anticoagulation
							• Single-level equivalent to multilevel shots
**ESP**	ESM-to-TP	Dorsal and ventral rami of spinal nerve roots	Yes	Ipsilateral hemithorax	Sternotomy (BLB)	20 mL at the 5th TP	• Bilevel-injection to improve LA spread
							• Preemptive approach
**PECS I**	PMAJOR-to-PMINOR	Medial and lateral pectoral nerves	No	Narrow upper anterolateral chest wall	Minimally invasive thoracotomy (ULB)	15 mL at the 3rd rib	• Inadequate for sternotomy
**PECS II**	PMAJOR-to-PMINOR (1) and SUPRA- or SUB-SAM (2)	PECS I, LTN and TDN	No	Wide upper anterolateral chest wall, including axilla	Minimally invasive thoracotomy (ULB)	30 mL at the 3rd rib	• Inadequate for sternotomy
							• Perform (1) after (2) with a single-pass approach
**SAPB**	SUPRA- or SUB-SAM	Lateral branches of ICN, including LTN and TDN with superficial SABP	No	Lateral chest wall	Minimally invasive thoracotomy (ULB)	30–40 mL at the 4th -5th rib	• Inadequate for sternotomy
							• Anterior spread with deep SAPB; posterior spread with superficial SAPB
**PIFB**	PMAJOR-to-EIM	Anterior branches of ICN	No	Parasternal	Sternotomy (BLB)	20 mL at the 4th rib	• Combined
**TTMPB**	INNIM-to-TTM	Anterior branches of ICN	No	Parasternal	Sternotomy (BLB)	20 mL at the 4th rib	• Combined

#—Single-shot blocks refer to one time LA injection without catheter placement. Depending on block technique, either single-level or multilevel LA deposition may be performed. Commonly used LA drugs are ropivacaine and bupivacaine with concentrations ranging from 0.25% to 0.5%. Maximum recommended doses are 2 mg/kg for bupivacaine and 3 mg/kg for ropivacaine, respectively [87]. PVB, paravertebral block; ESPB, erector spinae plane block; PECS I and II, pectoralis nerve blocks I and II; SAPB, serratus anterior plane block; PIFB, pecto-intercostal fascial plane block; TTMB, thoracic transversus plane block; TPVS, thoracic paravertebral space; ESM, erector spinae muscle; TP, thoracic transversus process; PMAJOR, pectoralis major muscle; PMINOR, pectoralis minor muscle; SAM, serratus anterior muscle; EIM, external intercostal muscle; INNIM, innermost intercostal muscle; TTM, thoracic transversus muscle; LTN, long thoracic nerve; TDN, thoracodorsal nerve; ICN, intercostal nerve; ULB, unilateral block; BLB, bilateral block.
